# FLT-PET-CT for the Detection of Disease Recurrence After Stereotactic Ablative Radiotherapy or Hyperfractionation for Thoracic Malignancy: A Prospective Pilot Study

**DOI:** 10.3389/fonc.2019.00467

**Published:** 2019-05-31

**Authors:** Susan M. Hiniker, Quaovi Sodji, Andrew Quon, Paulina M. Gutkin, Natasha Arksey, Edward E. Graves, Frederick T. Chin, Peter G. Maxim, Maximilian Diehn, Billy W. Loo

**Affiliations:** ^1^Department of Radiation Oncology, Stanford University School of Medicine, Stanford, CA, United States; ^2^Stanford Cancer Institute, Stanford University School of Medicine, Stanford, CA, United States; ^3^Department of Nuclear Medicine, Stanford University School of Medicine, Stanford, CA, United States; ^4^Department of Radiation Oncology, Indiana University, Indianapolis, IN, United States

**Keywords:** stereotactic ablative radiotherapy, 3-deoxy-3-[^18^F]-fluorothymidine, positron emission tomography, thoracic malignancy, disease recurrence

## Abstract

Differentiating local recurrence from post-treatment changes on PET scans following stereotactic ablative radiotherapy (SABR) or hyperfractionation for lung tumors is challenging. We performed a prospective pilot study of 3-deoxy-3-[^18^F]-fluorothymidine (FLT)-PET-CT in patients with equivocal post-radiation FDG-PET-CT to assess disease recurrence.

**Methods:** We prospectively enrolled 10 patients, 9 treated with SABR and 1 with hyperfractionated external beam radiotherapy for thoracic malignancy with subsequent equivocal follow-up FDG-PET-CT, to undergo FLT-PET-CT prior to biopsy or serial imaging. FLT-PET scans were interpreted by a radiologist with experience in reading FLT-PET-CT and blinded to the results of any subsequent biopsy or imaging.

**Results:** Of the 10 patients enrolled, 8 were evaluable after FLT-PET-CT. Based on the FLT-PET-CT, a blinded radiologist accurately predicted disease recurrence vs. inflammatory changes in 7 patients (87.5%). The combination of higher lesion SUV_max_ and higher ratio of lesion SUV_max_ to SUV_max_ of mediastinal blood pool was indicative of recurrence. Qualitative assessment of increased degree of focality of the lesion also appears to be indicative of disease recurrence.

**Conclusion:** Adjunctive FLT-PET-CT imaging can complement FDG-PET-CT scan in distinguishing post-treatment radiation changes from disease recurrence in thoracic malignancies. These findings support the investigation of FLT-PET-CT in a larger prospective study.

## Key Points

- QUESTION: Is FLT-PET-CT capable of distinguishing between disease recurrence and post-radiation changes after radiotherapy for thoracic malignancy?
- PERTINENT FINDINGS: In this prospective pilot study of 10 patients with thoracic malignancy treated with stereotactic ablative radiotherapy or hyperfractionated external beam radiotherapy and follow up PET/CT equivocal for disease recurrence, FLT-PET-CT accurately predicted disease recurrence or lack thereof in 7 of 8 patients evaluable (87.5%). Recurrent disease was associated with both a SUV_max_ >2.0 and ratio of SUV_max_ to mediastinal blood pool SUV_max_ > 2.0.
- IMPLICATIONS FOR PATIENT CARE: FLT-PET-CT can complement FDG-PET-CT in distinguishing post-radiotherapy changes from disease recurrence in thoracic malignancy, however, a larger prospective trial is needed.

## Introduction

Stereotactic ablative radiotherapy (SABR) is increasingly used for the definitive treatment of early-stage non-small cell lung cancer (NSCLC), as well as for oligometastatic lesions and other sites of disease including liver, pancreas, prostate (1–5). However, appropriate post-therapy follow-up imaging has not been well-established, with recommended surveillance regimens for early-stage NSCLC varying between current NCCN guidelines and recent RTOG studies (6). Compared to computed tomography (CT) which provides morphological information, positron emission tomography (PET) informs on tumor metabolism which may be more accurate in evaluating early response to therapy (7). [^18^F]-fluorodeoxyglucose-positron emission tomography (FDG-PET) has been an important tool in the evaluation of treatment response after radiotherapy, and metabolic activity on follow-up FDG-PET-CT in previously treated tumors has been used as a biomarker of response to treatment (8). As the [^18^F]-FDG tracer measures glucose metabolism which is upregulated in activated inflammatory cells, post-treatment inflammation can reduce the specificity of FDG-PET-CT (7, 9, 10). Indeed, SABR-induced lung injury can lead to a transient increase in FDG avidity due to influx of inflammatory cells, thus increasing the rate of false positive scans (11, 12). The difficulty interpreting FDG-PET-CT scans after SABR frequently results in unnecessary imaging and biopsy to evaluate local recurrence of disease (13).

3′-Deoxy-3′-[^18^F]-fluorothymidine (FLT)-PET-CT is a promising molecular imaging approach which measures tumor cell proliferation and has been utilized in various cancer types including lymphoma, leukemia, bone and soft tissue sarcomas, breast, head and neck, esophageal and lung cancers to assess treatment response (14). Upon uptake by cells, FLT is phosphorylated by thymidine kinase 1 (TK1), resulting in intracellular trapping. FLT uptake has subsequently been shown to reflect TK1 activity which has been correlated to cellular proliferation (15). Furthermore, tumor uptake of FLT been shown to be proportional to Ki-67 index (16). Unlike [^18^F]-FDG, [^18^F]-FLT does not appear to accumulate during inflammation but is an *in vivo* maker of cellular proliferation, thus enabling evaluation of tumor proliferation heterogeneity (7). Recent studies have indicated a potential role for FLT-PET-CT in assessment of response after conventionally fractionated radiotherapy (17, 18). The use of FLT-PET-CT after thoracic SABR has not been studied but may represent a useful tool in discriminating local recurrence from false-positive FDG-PET-CT signals.

**Figure 1:**
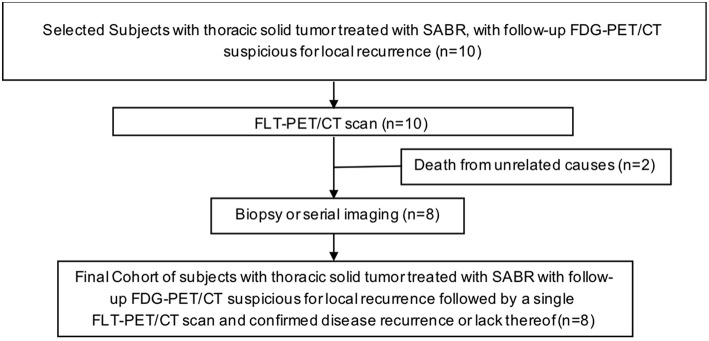
Scheme of the FLT-PET/CT prospective pilot study.

We sought to evaluate the utility of FLT-PET-CT in differentiating local recurrence from post-radiation changes in patients with equivocal FDG-PET-CT scans (see Figure 1 for study scheme). We hypothesized that FLT-PET-CT as a marker of cellular proliferation could add additional information in distinguishing recurrence from post-radiation changes.

## Materials and Methods

### Patients

We identified patients with thoracic malignancy treated with radiation at our institution. This study was approved by the Institutional Review Board, was performed in accordance to the principles of the Declaration of Helsinki and all subjects signed an informed consent. We collected and analyzed patient and treatment characteristics (Table 1), including gender, age, stage, history of chronic obstructive pulmonary disease (COPD), current or past history of smoking, radiotherapy details, imaging characteristics, and follow-up information. Inclusion criteria included a histological or cytological diagnosis of thoracic solid malignancy, age ≥18 years old and signing an informed consent. Pregnant patients were excluded from the study. Following treatment, patients are re-evaluated during regularly scheduled clinic visits. At our institution, patients are routinely followed in the clinic every 3 months for the first 2 years of follow-up, then every 4–6 months until year 5 with annual visits thereafter. Patients frequently undergo FDG-PET-CT scans every 3 months until the SUV of the treated lesion normalizes. If there are no other lesions of concern, patients are then followed with CT scans. Clinical-grade [^18^F]-FDG was supplied by the Cyclotron and Radiochemistry Facility (CRF) in the Molecular Imaging Program at Stanford University (MIPS). Ten patients with median age of 70 years (range 51–81) were enrolled on trial and underwent FLT-PET-CT after a suspicious surveillance FDG-PET-CT (Table 1).

**Table 1:** Patient and tumor characteristics.

**Characteristic**	***n***
**Sex**	
Male	6
Female	4
**Age (Years)**	
Median	70
Range	51–81
**Histology**	
Lung, adenocarcinoma	3
Lung, squamous cell adenocarcinoma	6
Metastatic colon adenocarcinoma	1
**Stage**	
I	7
IIIB	1
IV	2
**Radiation Treatment**	
SABR	
25 Gy in 1 fraction	2
50 Gy in 4 fractions	6
50 Gy in 5 fractions	1
Hyperfractionated EBRT	
60 Gy, 1.2 Gy/fraction B.I.D^*^	1

*All patients had equivocal follow-up FDG-PET scan, suspicious for recurrent disease*.

* *Reirradiation in patient with recurrent mediastinal lymphadenopathy. EBRT, External Beam Radiotherapy; SABR, Stereotactic Ablative Radiotherapy*.

### FLT-PET-CT

We proposed and designed a prospective pilot study investigating the utility of FLT-PET-CT in detecting tumor recurrence after thoracic radiation. We prospectively enrolled 10 patients who had a follow-up FDG-PET-CT scan interpreted as indeterminate for local recurrence following thoracic radiotherapy. Nine patients were treated with SABR, and one with hyperfractionated EBRT (external beam radiation therapy) due to reirradiation of recurrent mediastinal lymphadenopathy (Table 1). Patients underwent a single FLT-PET-CT scan for further characterization of the suspicious lesion prior to biopsy. If biopsy was not deemed safe, patients were followed with further surveillance imaging. No changes in management were made on the basis of the FLT-PET-CT. All PET scans were performed at the Stanford Department of Radiology, using the GE Discovery 690 PET-CT scanner with spatial resolution of 4.70 mm and 5.06 mm at 1 cm and 10 cm off axis respectively, and sensitivity of 7.5 cps/kBq (GE Healthcare, Chicago, Illinois) (19). Clinical-grade [^18^F]-FLT was synthesized in an automated module (TRACERlab, GE Healthcare) at the Cyclotron and Radiochemistry Facility in the Molecular Imaging Program at Stanford University as previously described under an Investigational New Drug protocol approved by the Food and Drug Administration (20). Each patient received 185±37 MBq of [^18^F]-FLT and a molar activity of 78.44 ± 52.91 GBq/μmol at the time of injection (molar activity at the end of synthesis of 157.99 ± 91.76 GBq/μmol) 1 h before the single PET-CT scan. The FLT-PET-CT scan was interpreted by a radiologist familiar with interpretation of FLT-PET scans and also blinded to the results of the biopsy and/or subsequent imaging. Interpretation criteria included a semi-quantitative assessment of the SUV_max_ of the lesion and background activity in the lung and mediastinum, as well as a qualitative assessment of the focality of the lesion.

### Statistical Analysis

Only descriptive statistics are provided as this is a pilot study.

## Results

**Figure 2:**
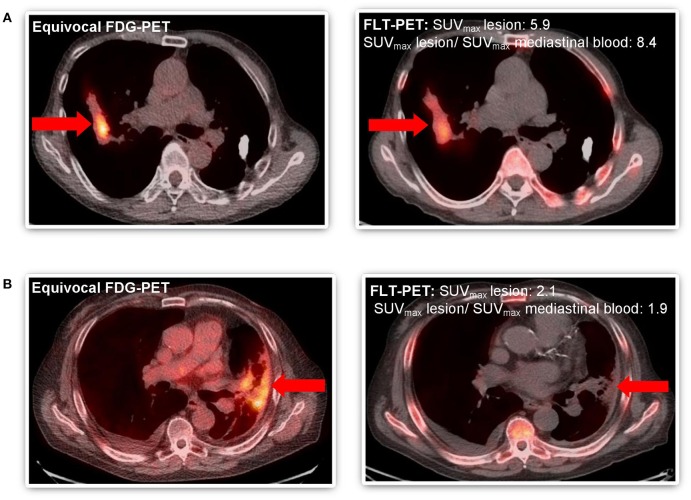
Comparative imaging of FDG-PET and FLT-PET in patients with equivocal follow-up FDG-PET scan after SABR to thoracic malignancy. **(A)** Patient #4 WITH disease recurrence predicted by FLT-PET and confirmed by progression on subsequent serial imaging (both SUV_max_lesion >2.0 and ratio SUV_max_ lesion and mediastinal blood pool >2.0) and focal FLT uptake. **(B)** Patient #8 WITHOUT disease recurrence predicted by FLT-PET and confirmed by biopsy (SUV_max_lesion >2.0 but ratio SUV_max_ lesion and mediastinal blood pool < 2.0). Equivocal lesion by FDG-PET highlighted by arrow (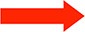).

Among our cohort, no toxicities related to [^18^F]-FLT administration and PET-CT scans were noted. Only descriptive statistics are provided. Two patients died of unrelated causes before final determination of disease recurrence could occur. Of the remaining 8 patients, 5 underwent biopsy, and 3 were deemed unsuitable for biopsy and were followed with serial imaging until unequivocal progression or resolution of the area of concern. Five patients out of 8 (63%) had disease recurrence confirmed by biopsy or serial imaging. Among the 5 patients with recurrent disease, 4 had their FLT-PET-CT scans interpreted as positive for recurrent disease (Table 2). The fifth patient had FLT-PET scan interpreted as negative, but biopsy revealed recurrent disease. Three patients had findings interpreted as negative for recurrence on FLT-PET-CT scan and the absence of recurrence subsequently confirmed by biopsy or serial imaging (Table 2). Representative images from equivocal FDG-PET-CT scans and corresponding FLT-PET-CT scans are shown in Figure 2. The FLT-PET-CT scan accurately predicted recurrent disease or lack thereof in 7 of 8 patients (87.5%) (Figure 2 and Table 2). Although final disease status could not be achieved in 2 of the patients enrolled in this trial, the treated lesion remained stable in size and FDG avidity on a follow-up FDG-PET-CT scan performed 4 months after FLT-PET-CT in 1 of the patient (Table 2).

**Table 2:** FLT-PET-CT scan and confirmation of disease recurrence.

**Patient**	**SUV_**max**_ lesion-FDG**	**SUV_**max**_ lesion-FLT**	**SUV mediastinal blood pool-FLT**	**Ratio of SUV_**max**_ lesion-FLT and SUV_**max**_ mediastinal blood pool-FLT**	**Focality of lesion on FLT**	Recurrence prediction based on radiologist interpretation of FLT^**^	**Recurrence confirmation by biopsy or serial imaging**
1	3.4	3.9	1.0	3.9	No	Recurrence	Recurrence^*^
2	10	3.7	0.8	4.6	Yes	Recurrence	Recurrence^*^
3	6	2.0	1.6	1.3	No	No Recurrence	Recurrence^*^
4	6.8	5.9	0.7	8.4	Yes	Recurrence	Recurrence^†^
5	4.2	1.7	1.0	1.7	No	No Recurrence	N/A
6	2.4	1.3	0.9	1.4	No	No Recurrence	No Recurrence^†^
7	4.1	1.6	1.2	1.3	No	No Recurrence	N/A^a^
8	3.6	2.1	1.1	1.9	No	No Recurrence	No Recurrence^*^
9	6.0	2.8	0.7	4.0	Yes	Recurrence	Recurrence^*^
10	4.2	1.5	0.6	2.5	No	No Recurrence	No Recurrence^†^

*FLT-PET correctly predicted recurrence status in 7 of 8 assessable cases. Combination of both lesion FLT SUV_max_ > 2.0 plus ratio of lesion FLT SUV_max_ and mediastinal blood pool FLT SUV_max_ > 2.0 is indicative of disease recurrence*.

* Biopsy;

† serial imaging;

** *radiologist interpreting FLT-PET scans was blinded to biopsy results and subsequent imaging. N/A: Patient died of unrelated causes before final determination of disease status could be achieved by biopsy or serial imaging*.

a *Although final determination of disease status could not be achieved, lesion remained stable in size and FDG avidity on follow-up FDG-PET scan, 4 months after FLT-PET-CT*.

On exploratory analysis, among the 4 patients for whom the FLT-PET-CT scan accurately predicted disease recurrence, both lesion SUV_max_ and the ratio of lesion SUV_max_ to mediastinal blood pool SUV_max_ were >2.0. Among the 3 patients accurately predicted not to have recurrent disease, either the lesion SUV_max_ and/or the ratio of lesion SUV_max_ to mediastinal blood pool SUV_max_ was < 2.0. In addition, the qualitative assessment of focality on FLT-PET-CT was also indicative of disease recurrence, as all three patients with focal FLT uptake had recurrent disease confirmed by biopsy or serial imaging.

## Discussion

There is increasing interest in the use of FLT-PET-CT in the evaluation of various malignancies (7). In this study, we sought to evaluate the role of FLT-PET-CT in distinguishing disease recurrence from SABR-induced inflammation. FLT-PET-CT is perhaps most widely used in the setting of lymphoma, where very early changes in [^18^F]-FLT uptake following chemotherapy have been correlated with treatment response (21). In the setting of NSCLC, the role of FLT-PET-CT has been studied after carbon-ion radiotherapy, and found to be feasible for evaluating treatment response. However, the presence of radiation pneumonitis interfered with evaluation of post-treatment response (22). In lung cancer patients treated with conventionally fractionated radiotherapy, radiation-induced pneumonitis interfered with assessment of metabolic tumor response by FDG-PET-CT, however FLT-PET-CT accurately predicted patients with local tumor control and recurrence (17). FLT may also have the ability to evaluate early response to therapy during the radiation course. A study of 12 patients with NSCLC treated with radiation alone evaluated the changes in FLT-PET-CT during the radiation course, and found an early significant decrease in primary tumor SUV_max_ and mean by 25% after 5–11 fractions of radiation, suggesting a role for FLT-PET-CT in the evaluation of early response to radiotherapy (18).

Preclinical data demonstrates the ability of FLT-PET-CT to differentiate between inflammatory and malignant lesions in settings of equivocal FDG-PET-CT (23). Indeed, in our study we did not see significant [^18^F]-FLT uptake in the setting of apparent radiation pneumonitis (Figure 2, patient 8). This increased tumor-specificity relative to FDG-PET-CT will be advantageous in response assessment following treatment, especially early on when inflammatory changes have not yet subsided, or following RT or combined modality therapy where post-therapy inflammatory changes may be observed for 2–3 months or longer. The lack of [^18^F]-FLT uptake does not always correlate with absence of recurrence, as seen in patient #3. This may be related to reliance on the *de novo* thymidine synthesis in some tumors, as opposed to the thymidine salvage pathway in which TK1 is involved (24).

Our data suggests that FLT-PET-CT may provide an additional non-invasive benefit in the detection of disease recurrence after thoracic SABR. Higher lesion SUV_max_ and higher ratio of the lesion SUV_max_ to the mediastinal blood pool SUV_max_ appears to be associated with disease recurrence. Given the small number of patients in this study, no conclusions can be drawn regarding SUVmax cut-off values and association with recurrence. Few studies have examined SUV_max_ values in FLT-PET-CT scans performed as follow-up imaging for patients with NSCLC after completion of radiation. In concordance with our findings, analysis of a series of 22 patients treated with conventionally fractionated EBRT for NSCLC found a trend to higher lesion SUVmax in recurrent tumors, with mean SUVmax of 1.7 among patients with local control and 2.8 among patients with tumor recurrence (17). FLT-PET-CT may also have a role in predicting ultimate outcome after treatment and in early response detection in NSCLC. Among patients with NSCLC treated with carbon ion radiotherapy, higher pre-treatment SUVmax on FLT-PET-CT was found to be significantly correlated with recurrence (21). Others have described a decrease in SUVmax of the primary lung tumor on FLT-PET-CT reflecting early response to therapy as soon as after 1–2 weeks of conventionally fractionated EBRT (18, 25). Focality of [^18^F]-FLT uptake may also provide an additional measure of evaluation of tumor recurrence. However, while FLT-PET-CT provide several advantages over FDG-PET-CT in response assessment, it has limits as a biomarker of cellular proliferation. Factors which can interfere with [^18^F]-FLT delivery, intracellular transport via nucleoside transporters and intracellular trapping can compromise FLT-PET-CT. Furthermore, FLT, a surrogate of tumor proliferation as a function of thymidine salvage pathway may not accurately reflect proliferation in malignancies relying on the thymidine *de novo* synthesis (24).

## Conclusion

In summary, adjunctive FLT-PET-CT can complement FDG-PET-CT in distinguishing post-SABR changes from disease recurrence in thoracic malignancy. Our findings argue for a larger prospective study to evaluate the role of FLT-PET-CT in response assessment after SABR for thoracic malignancies.

## Ethics Statement

This study was approved by the Institutional Review Board, was performed in accordance to the principles of the Declaration of Helsinki and all subjects signed an informed consent.

## Author Contributions

SH and QS contributed to the conceptualization, data curation, investigation, methodology, project administration, validation, visualization, writing, review, and editing. AQ analyzed, interpreted, and collected patient information, and was a major contributor in coordinated writing of this manuscript. PG contributed to the data curation, project administration, resources, writing, review, and editing. NA, EG, FC, and PM contributed to the data curation, formal analysis, investigation, methodology, resources, software,validation, visualization, writing-review, and editing. MD and BL contributed to the conceptualization, investigation, methodology, project administration, resources, supervision, validation, visualization, and writing-review, and editing of this manuscript.

### Conflict of Interest Statement

BL, MD, EG, and PM have received research support from Varian Medical Systems. BL is a board member of TibaRay. The remaining authors declare that the research was conducted in the absence of any commercial or financial relationships that could be construed as a potential conflict of interest.
